# Metabolomic Assessment of Induced and Activated Chemical Defence in the Invasive Red Alga *Gracilaria vermiculophylla*

**DOI:** 10.1371/journal.pone.0029359

**Published:** 2011-12-21

**Authors:** Göran M. Nylund, Florian Weinberger, Martin Rempt, Georg Pohnert

**Affiliations:** 1 Institute for Inorganic and Analytical Chemistry, Instrumental Analytics/Bioorganic Analytics, Friedrich-Schiller-University Jena, Jena, Germany; 2 Leibniz-Institut für Meereswissenschaften (IFM-GEOMAR), Kiel, Germany; East Carolina University, United States of America

## Abstract

In comparison with terrestrial plants the mechanistic knowledge of chemical defences is poor for marine macroalgae. This restricts our understanding in the chemically mediated interactions that take place between algae and other organisms. Technical advances such as metabolomics, however, enable new approaches towards the characterisation of the chemically mediated interactions of organisms with their environment. We address defence responses in the red alga *Gracilaria vermiculophylla* using mass spectrometry based metabolomics in combination with bioassays. Being invasive in the north Atlantic this alga is likely to possess chemical defences according to the prediction that well-defended exotics are most likely to become successful invaders in systems dominated by generalist grazers, such as marine macroalgal communities. We investigated the effect of intense herbivore feeding and simulated herbivory by mechanical wounding of the algae. Both processes led to similar changes in the metabolic profile. Feeding experiments with the generalist isopod grazer *Idotea baltica* showed that mechanical wounding caused a significant increase in grazer resistance. Structure elucidation of the metabolites of which some were up-regulated more than 100 times in the wounded tissue, revealed known and novel eicosanoids as major components. Among these were prostaglandins, hydroxylated fatty acids and arachidonic acid derived conjugated lactones. Bioassays with pure metabolites showed that these eicosanoids are part of the innate defence system of macroalgae, similarly to animal systems. In accordance with an induced defence mechanism application of extracts from wounded tissue caused a significant increase in grazer resistance and the up-regulation of other pathways than in the activated defence. Thus, this study suggests that *G. vermiculophylla* chemically deters herbivory by two lines of defence, a rapid wound-activated process followed by a slower inducible defence. By unravelling involved pathways using metabolomics this work contributes significantly to the understanding of activated and inducible defences for marine macroalgae.

## Introduction

An extensive part of chemical ecology research focuses on plant-herbivore interactions. Both terrestrial plants and marine macroalgae produce a rich variety of secondary metabolites that can function as defences against herbivores [1], [2]. These defence metabolites can be constitutively expressed or induced, whereby the production is increased in response to the attack. The occurrence of inducible chemical defences following herbivory, or mechanical simulation of herbivory is well documented for a large number of terrestrial plants [3]. Fewer examples exist for marine macroalgae, but recent research suggests that induced resistance may also be common among them [4]–[6]. Another dynamic response in plants is the activated defence, whereby pre-formed metabolites are rapidly transformed into more reactive and effective substances after tissue disruption [7]. This process is common in terrestrial plants [8], and has also been observed for macroalgae [5], [9], [10]. Activated defences resemble inducible defences in that they need to be stimulated, but are more similar to constitutive defences in that the response time to herbivory is very rapid and resources are invested in the precursors of defence metabolites before the injury occurs [3], [7]. Activated strategies might be selected over constitutive defences to protect the plant from autotoxicity [11], [12] or to avoid attraction of specialist herbivores cuing on the defensive chemicals [13]–[15]. Inducible and activated defences can be stimulated by mechanical damage of tissue, exposure to herbivore-derived elicitors, or a combination of both [3], [16]. In comparison to terrestrial plants, however, the mechanistic knowledge of chemical defences is poor for marine macroalgae. This restricts our understanding in the chemically mediated interactions that take place between algae and other organisms.

In many cases, chemical ecologists have shown defence roles of previously described plant metabolites by testing their effect on palatability and grazer performance [17]–[19]. Alternatively, defence metabolites have been identified by bioassay-guided fractionation, whereby active extracts are chromatographically separated and the fractions are tested for activity. Bioactive metabolites are thereby purified in an iterative way until the metabolite(s) responsible for the biological effect can be identified [20], [21]. Although these approaches resulted in comprehensive knowledge of the chemical mediation of plant-herbivore interactions there are limitations like the time consuming fractionation and the requirements for single active metabolites that excludes the identification of mixtures where compounds exhibit synergistic activity. However, recent developments in metabolomics enable new approaches towards the characterisation of the chemically mediated interactions of organisms with their environment [22]. Particularly in chemical ecology research, where often metabolic responses to stimuli like herbivory or mechanical tissue disruption are relevant, metabolomic techniques have the potential to be an important complement to traditionally used methods [23]. Using metabolomic techniques secondary metabolites produced or transformed during plant herbivore interactions can be recognized and constitutively expressed metabolites can easily be excluded from further analysis. Thereby the task of identifying relevant metabolites in e.g. defence responses is eased.

In this study we use metabolomic techniques based on liquid chromatography-mass spectrometry (LC/MS) and gas chromatography-mass spectrometry (GC/MS) to profile the metabolic response of the red alga *Gracilaria vermiculophylla* (synonym *G. asiatica*) against herbivory. *G. vermiculophylla* is a perennial macroalga native to the Northwest Pacific [24]. During the last two decades this alga has been detected at the western and eastern coast of the Atlantic and recently also to the Kattegat and the western Baltic [25]–[28]. This species spreads continuously and invasively changes the local flora and fauna. It has been predicted that well-defended exotics are most likely to become successful invaders in systems, such as marine benthic communities, where generalist consumers exert a significant selection pressure [29]. Previous feeding trials show that in the western Baltic *G. vermiculophylla* is low preferred by generalist herbivores, which may indicate the presence of defence mechanisms against herbivory [30]. Here we present results from an explorative search for possible induced and activated chemical defence metabolites in this red alga using metabolomics and bioassays with the mesoherbivore *Idotea baltica*, an important and abundant generalist grazer in the Baltic Sea [31], [32]. Specifically we asked whether (1) simulation of herbivory by mechanical wounding elicits an activated defence, (2) mechanical wounding causes a similar metabolic response as natural grazing, (3) defence can be induced by signals from attacked tissue, and (4) local mechanical wounding leads to an activated defence reaction in neighbouring tissue in *G. vermiculophylla* (Table 1)? A particular focus was to elucidate the biochemical pathway involved in the activated defence.

**Table 1:** Questions asked and methods used to study chemical defences in *Gracilaria vermiculophylla*.

Question	Method
Does simulation of herbivory by mechanical wounding elicit an activated defence?	LC/MS and GC/MS based metabolomics to study metabolic responses in mechanically wounded tissue. Resulted grazer deterrence assessed by *Idotea baltica* bioassays.
Follow-up question: Are up-regulated metabolites in the activated defence produced de-novo from arachidonic acid?	LC/MS analysis of labelling pattern following administration of deuterated arachidonic acid to mechanically wounded tissue.
Does mechanical wounding cause a similar metabolic response as natural grazing?	Exposure of algae to 1 and 2 h intense grazing by *Idotea baltica* followed by targeted analysis of the metabolites significantly up-and down regulated in response to mechanical wounding.
Can a defence be induced by signals from attacked tissue?	LC/MS and GC/MS based metabolomics to study metabolic responses in intact tissue exposed to extracts from mechanically wounded algae. Resulted grazer deterrence assessed by *Idotea baltica* bioassays.
Does local mechanical wounding lead to an activated defence reaction in neighbouring tissue?	Local mechanical wounding of tissue and extraction of intact neighbouring tissue for targeted analysis of the metabolites significantly up-and down regulated in response to mechanical wounding.

## Results

### Wounding of algal tissue - effects on palatability and metabolic profile

**Figure 1:**
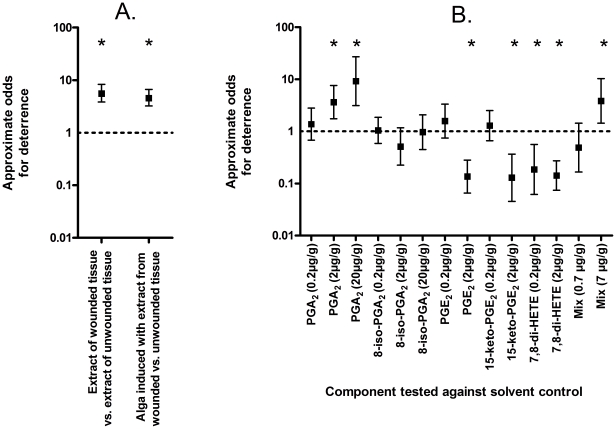
Feeding preference of *Idotea baltica*. Odds ω for the relative deterrence of the herbivore by artificial diets containing either algal extracts or biomass (A) or isolated eicosanoids (B) are given. Means±95 % confidence intervals are given, asterisks indicate odds that are significantly above or below 1 (Fisher's exact test, p<0.05). A: extract from mechanically wounded algae was tested relative to controls containing extract from intact algae and tissue of algae induced with extract from mechanically wounded algae was tested relative to tissue of control algae that were treated with extract from intact algae. B: five different eicosanoids were tested relative to solvent controls, either isolated or in combination (PGA_2_:PGE_2_:15-keto-PGE_2_:7,8-de-HETE in a ratio of 1∶1∶1∶0.5).

**Figure 2:**
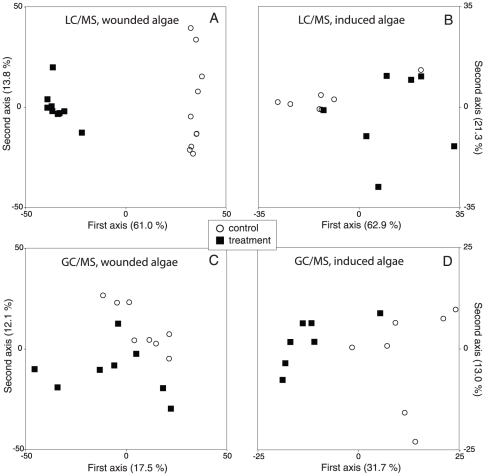
LC/MS and GC/MS metabolic profiling. Principal coordinate analysis of RT- *m/z* pairs derived from LC/MS and GC/MS analyses of *Gracilaria vermiculophylla* extracts. A & C: Extracts from mechanically wounded algae and controls, analysed by LC/MS and GC/MS, respectively. B & D: Extracts from algae exposed to extracts from previously mechanically wounded and control tissue, analysed by LC/MS and GC/MS, respectively. Numbers in brackets are the percentages of original variance explained by the derived axes.

**Figure 3:**
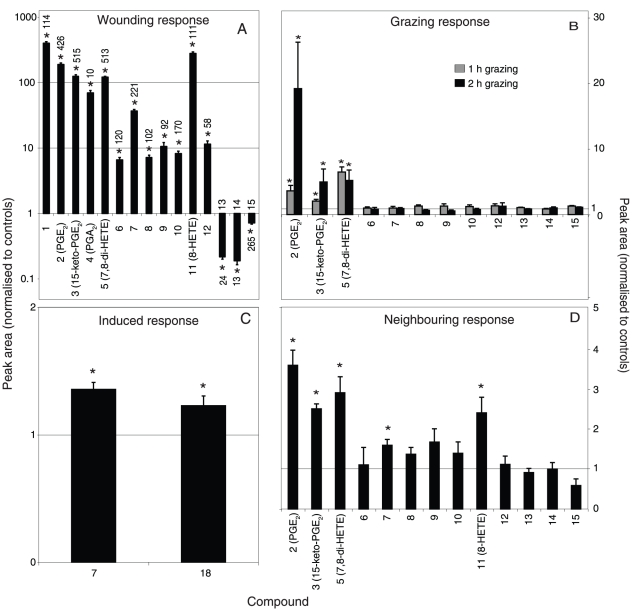
Metabolic response of *Gracilaria vermiculophylla* analysed by LC/MS. A: metabolites up- and down-regulated followed by mechanical simulation of herbivory. B: effects of *Idotea baltica* feeding on the expression of metabolites 2 (PGE_2_), 3 (15-keto-PGE_2_), 5 (7,8-di-HETE), 6 - 10 and 12-15. C: metabolites up-regulated followed by exposure to extracts from mechanically wounded algae. D: neighbouring effects of local mechanical wounding on the expression of metabolites 2 (PGE_2_), 3 (15-keto-PGE_2_), 5 (7,8-di-HETE), 6–10, 11 (8-HETE) and 12–15. Metabolic responses to the treatments are shown as peak areas normalised to corresponding controls. *Significant difference from the control (see the text for details). Data are means+SE, n = 10 (A), 8 (B, D) or 7 (C). Numbers at the end of the bars in A are the relative peak area for the metabolites up and down-regulated followed by simulation of herbivory. Note logaritmic scale in A.

**Figure 4:**
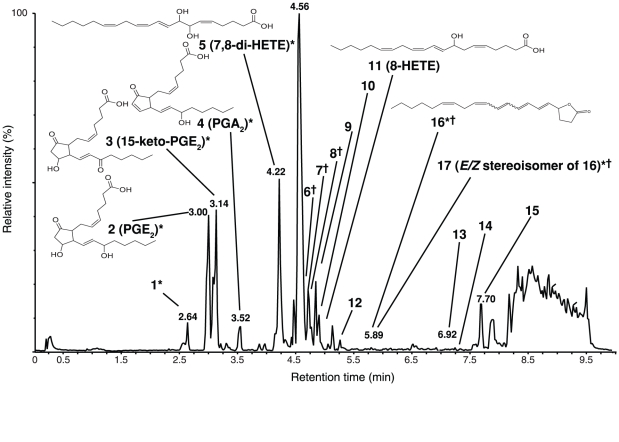
LC/MS chromatogram of mechanically wounded *Gracilaria vermiculophylla*. * Indicates de novo biosynthesis after labelled arachidonic acid administration with incorporation rates of 43.2–62.4 %. † Indicates co-eluting peaks.

In a 2-way choice feeding experiment where the mesoherbivore *I. baltica* was offered food containing extracts from mechanically wounded and intact *G. vermiculophylla*, it consumed significantly more of controls containing extracts from intact tissue (ω>1; Fisher's exact test, p<0.001; Fig. 1A). This increase in grazer resistance was accompanied by a significant change in the metabolic profile of the alga (LC/MS data: 1-factor PERMANOVA, *F*_1,18_ = 123.14, p<0.0001; GC/MS data: 1-factor PERMANOVA, *F*_1,14_ = 6.54, p = 0.0011; Fig. 2A & C). Analysis of LC/MS data using CAP showed that 11 metabolites were significantly up-regulated and 3 significantly down-regulated in the extracts from mechanically wounded algae compared to the controls (Fig. 3A). Two of the up-regulated metabolites were identified as 8-HETE and 7,8-di-HETE (metabolite No. 5 and 11 Table 2, Fig. 4), by comparison to known standards [33]. The prostaglandins PGE_2_ and 15-keto-PGE_2_ (metabolites No. 2 and 3 Table 2, Fig. 4) were identified by comparison of their ^1^H- and ^13^C-NMR data to the literature as well as co-injection with commercially available standards. Inspection of the LC/MS chromatograms revealed a minor arachidonic acid derived metabolite that was not detected by the chemometric analysis. This metabolite was significantly up-regulated in the extracts from mechanically wounded algae compared to the controls (t-test, *t* = 13.89, p<0.0001; metabolite No. 4 Table 2, Fig. 3A) and comparison of its ^1^H- and ^13^C-NMR data to the literature as well as co-injection with a commercially available standard identified it as the prostaglandin PGA_2_ (Table 2, Fig. 4). The other up-regulated metabolites were not identified. Two compounds that were detected from inspection of UV spectra were selected for further structure elucidation (Fig. 4 metabolite No. 16 and 17). The metabolites in question were only present in the extracts from mechanically wounded algae and NMR analysis revealed the structures of isomers of a conjugated lactone (No. 16 in Fig. 4, Rempt et al., submitted). The three prostaglandins as well as the 8-HETE, the 7,8-di-HETE and one unidentified metabolite (No. 7) were 70 to 400 times up-regulated in the mechanically wounded tissue (Fig. 3A). The most pronounced regulated metabolites were all derived de novo from arachidonic acid, as could be proven by analysis of the labelling pattern after administration of deuterated precursors (Table 2, Fig. 4).

**Table 2:** Identified compounds with LC-UV/Vis-MS.

Label	Retention time (min)	Highest significant m/z (ESI-negative)	Identity	Deuterated arachidonic acid incorporation rate (%)	Natural concentration (µg g^−1^ wet weight)
1	2.64	431	arachidonic acid derived	53.8	-
2	3.00	351	**PGE_2_**	88.5	2.1±0.8
3	3.14	349	**15-keto-PGE_2_**	104.1	7.9±4.2
4	3.52	333	**PGA_2_**	70.0	0.6±0.4
5	4.22	335	**7,8-di-HETE**	110.1	2.4±0.9
6	4.47	589	not identified	0	-
7	4.56	555	not identified	0	-
8	4.62	699	not identified	0	-
9	4.75	483	not identified	0	-
10	4.85	585	not identified	0	-
11	4.90	319	**8-HETE**	77.8	0.38±0.11
12	5.13	537	not identified	0	-
13	6.92	814	possible phospholipd	0	-
14	7.13	816	possible phospholipd	0	-
15	7.70	872	possible phospholipd	0	-
16 and 17	5.89	301 (ESI-pos)	**conjugated lactone**	56.4	9.8±3.2

Data showing natural concentrations of up-regulated metabolites are means±SD, n = 3. Incorporation rates are based on the ratio of the molecular ion of a metabolite and the molecular ion of the corresponding labelled metabolite. Bold entries were verified with authentic standards.

Analysis of GC/MS data using CAP identified 8 metabolites significantly up-regulated in the mechanically wounded algae (Table 3A). Fragment pattern analysis and comparison of obtained mass spectra with NIST library entries identified four of the metabolites, oleic acid and three oxylipins (7,8-di-HETE, PGE_2_, 15-keto-PGE_2_) also detected by the analysis of LC/MS data (Table 3A).

**Table 3:** Metabolic response of *Gracilaria vermiculophylla analysed by GC/MS.*

	Label	Retention time (min)	Base peak^1^	Suggested structure^2^	Peak area relative to controls
**A**	A	6.28	131		1.4±0.1
	B	9.25	129		12.2±3.1
	C	13.15	243		14.7±3.0
	D	15.22	339	**oleic acid**	2.2±0.5
	E	17.46	239		9.9±1.9
	F	17.69	287	**7,8-di-HETE**	62.1±22.7
	G	18.04	436	**15-keto-PGE_2_**	2.3±0.6
	H	18.27	225	**PGE_2_**	2.2±0.6
**B**	1	6.13	174	n-butylamine	1.8±0.1
	2	6.17	116		1.5±0.1
	3	7.03	210		1.1±0.0
	4	7.50	117		1.1±0.0
	5	7.73	299	phosphoric acid	1.4±0.1
	6	8.08	174	**glycine**	1.7±0.2
	7	8.52	204	**serine**	1.9±0.2
	8	8.73	218	**threonine**	1.2±0.0
	9	9.35	350		1.2±0.1
	10	9.52	234	saccharide	1.4±0.1
	11	9.62	233	malic acid	1.5±0.1
	12	9.97	156	pyroglutamic acid	1.4±0.1
	13	10.19	292	rythroric acid	1.2±0.1
	14	10.35	198		1.7±0.1
	15	10.67	142		1.5±0.1
	16	10.70	246	**glutamine**	1.7±0.1
	17	10.90	315		1.7±0.4
	18	11.75	299	glycerophosphate	1.8±0.2
	19	11.85	319		1.8±0.1
	20	11.89	156		1.7±0.2
	21	11.95	392		1.6±0.1
	22	12.17	273	citric acid	1.2±0.0
	23	12.19	142	ornithine	1.2±0.0
	24	12.24	157		1.5±0.0
	25	12.30	287	methyl citric acid	1.4±0.1
	26	12.40	173		1.6±0.1
	27	12.47	117	**myristic acid**	1.3±0.0
	28	12.70	319	monosaccharide	1.8±0.1
	29	12.90	260		1.4±0.1
	30	13.05	218		1.6±0.2
	31	13.27	319	monosaccharide	1.4±0.1
	32	13.70	319	monosaccharide	1.8±0.1
	33	13.79	313	**palmitic acid**	1.3±0.0
	34	14.02	217	myo-inositol	1.2±0.1
	35	14.59	319	monosaccharide	2.3±0.2
	36	15.74	117	**arachidonic acid**	1.6±0.1
	37	16.03	527		1.5±0.1
	38	16.86	371	**monopalmitin glycerol**	1.5±0.2
	39	16.94	283		1.1±0.1
	40	17.18	572		2.0±0.2
	41	17.26	557		1.6±0.2
	42	17.64	361	disaccharide	5.9±0.8
	43	19.83	204	saccharide	2.5±0.2
	44	20.36	129	**cholesterol**	1.4±0.1

A: metabolites significantly up-regulated followed by mechanical simulation of herbivory. B: metabolites significantly up-regulated followed by exposure to extracts from previously mechanically wounded algae. Metabolic responses are presented as peak areas normalised to corresponding controls (means±SE, n = 8 (A), or 7 (B)). Suggested structures are from comparisons of MS spectral data with spectra in the NIST library. Metabolites with confirmed structure by comparisons to known standards in bold.

1 The most intense ion is given (the ion *m/z* = 73 in not considered in this listing).

2 Library suggestion are only included if the match and reverse match were more than 790.

Natural grazing of *G. vermiculophylla* by *I. baltica* caused a comparable metabolic response as mechanical simulation of herbivory (Fig. 3A & B). Analysis of LC/MS data showed that 1 and 2 h grazing significantly changed the concentration of metabolites that were also effected in mechanically wounded tissue (1 h grazing: 1-factor PERMANOVA, *F*_1,14_ = 3.25, p = 0.021; 2 h grazing: 1-factor PERMANOVA, *F*_1,14_ = 3.74, p = 0.0038). Further analysis using CAP showed that PGE_2_ (2), 15-keto-PGE_2_ (3) prostaglandins and 7,8-di-HETE (5) were significantly up-regulated in tissue exposed to grazing for both 1 and 2 h, compared to controls (Fig. 3B). These were the same metabolites as 3 of the 5 most up-regulated ones in the mechanically wounded tissue (Fig. 3A & B). The consumption of algal tissue by *I. baltica* varied between 5 and 30 % of initial material.

### Induction experiment - effects on palatability and metabolic profile

In a 2-way choice feeding experiment where *I. baltica* was offered food made of *G. vermiculophylla* previously exposed to extract from mechanically wounded or intact algae, it consumed significantly more of the food derived from intact algal treated samples (ω>1; Fisher's exact test, p<0.001; Fig. 1A). This decrease in palatability was accompanied by a significant change in the metabolic profile of the alga (GC/MS data: 1-factor PERMANOVA, *F*_1,12_ = 13.65, p = 0.0009; Fig. 2D). Analysis of GC/MS data using CAP showed that 44 metabolites were significantly up-regulated (Table 3B). Comparisons of spectral data with spectra in the NIST library resulted in 26 tentative identifications of the metabolites and 9 of them were verified by comparisons to known standards (Table 3B). PERMANOVA showed no significant change in the metabolic profile of LC/MS detected metabolites (*F*_1,12_ = 2.86, p = 0.062; Fig. 2B), although further analysis using CAP showed that two metabolites were significantly up-regulated in tissue previously exposed to extract from mechanically wounded algae (Fig. 3C). MS fragment pattern analysis showed that one of the metabolites was identical to metabolite No. 6 from the mechanically wounded algae. The metabolites detected by LC/MS and GC/MS were only slightly up-regulated (less than 2 times) in the algae previously exposed to extract from mechanically wounded tissue with one exception: metabolite No. 42 detected by GC/MS was up-regulated 6 times (Fig. 3C, Table 3B).

### Neighbouring effect of local wounding

Local mechanical wounding of *G. vermiculophylla* tissue caused a metabolic response in neighbouring intact tissue that was comparable to the response to mechanical simulation of herbivory (Fig. 3D). Analysis of LC/MS data showed that this treatment significantly changed the abundance of metabolites that were also effected in mechanically wounded tissue (1-factor PERMANOVA, *F*_1,14_ = 5.92, p = 0.0007). Further analysis using CAP showed that again the two prostaglandins PGE_2_ (2), 15-keto-PGE_2_ (3), the 7,8-di-HETE (5), 8-HETE (11) and an unidentified metabolite (7) were significantly up-regulated in the undamaged tissue removed from filaments with damaged tips (Fig. 3D). These were the same metabolites as 5 of the 6 most up-regulated ones in the mechanically wounded tissue (Fig. 3A & D). The relative abundance of the other metabolites (6, 8 to 10 and 12 to 15) was not significantly different between treatments and controls.

### Natural concentration and biological activity of up-regulated metabolites

We tested available up-regulated metabolites in feeding assays with *I. baltica* in concentrations close to their natural abundance in wounded tissue (Table 2) and in elevated concentrations. The concentrations were selected to simulate on the one hand the concentration in the entire wounded tissue but also to evaluate the effect of potential high local concentrations around feeding sites. This procedure also allowed generation of structure activity profiles. Of the 5 tested metabolites (three naturally occurring prostaglandins, an isomer of PGA_2_ that is not found in *G. vermiculophylla* and the 7,8-di-HETE) only PGA_2_ exhibited a significant inhibitory activity (ω>1; Fisher's exact test, p = 0.001; Fig. 1B). PGE_2_, 15-keto-PGE_2_ and the di-HETE made food rather more attractive to the herbivore (Fig. 1B). If a mix of the oxylipins was administered in close to natural concentrations a significant inhibition of feeding activity was observed (ω>1; Fisher's exact test, p = 0.01).

## Discussion

Generally, the understanding of mechanisms in activated and inducible defences is poor for marine macroalgae in comparison with terrestrial plants, where defence pathways involving receptors for chemical signals of the herbivores, hormonal cascades and up-regulated biosynthetic machineries are rather well understood [34]–[36]. Most studies on macroalgae have tested the effect of direct grazing or artificial damage on palatability to herbivores [9], [37], and only a few have focused on identifying the defence metabolites or the elicitors of the defence response [7], [16], [38], [39]. In this study we used a metabolomics approach to investigate the nature of metabolites causing the increased resistance against herbivory, observed in mechanically wounded tissue and intact algae exposed to extracts from such tissue, and underlying metabolic pathways. The metabolomic measurements and the choice feeding experiments in this study show that *G. vermiculophylla* can rapidly respond to mechanical simulation of herbivory by a significant and substantial change in the metabolic profile (Fig. 2A) that leads to increased resistance against grazing by the isopod *I. baltica* (Fig. 1A). These metabolic changes cannot only be elicited by mechanical wounding in vitro; they are also observed after intense herbivory of *I. baltica* (Fig. 3B). Interestingly, not only direct mechanical wounding but also wounding of neighbouring tissue leads to the metabolic defence responses (Fig. 3D). In addition to these two rapid responses, signals released after mechanical wounding of algae also induce a slower defence response in *G. vermiculophylla* since exposure of intact algae to extract from mechanically wounded tissue resulted in increased resistance against grazing (Fig. 1B) and metabolic changes (Fig. 2D, Table 3B). Altogether, the results of this study suggest that *G. vermiculophylla* chemically deters herbivory by two lines of defence: a rapid wound-activated response that is followed by an inducible defence. The palatability of algae and algal extracts were assessed with bioassays using the crustacean *I. baltica*. This mesoherbivore species is an important and abundant generalist grazer [31], [32] and the main feeding enemy of *G. vermiculophylla* in the Baltic [30]. The results have thus a direct implication for the evaluation of the defence of the invasive alga.

The most pronounced metabolic changes detected in LC/MS experiments include the up-regulation of arachidonic acid derived oxylipins. Several eicosanoids, such as the prostaglandins PGE_2_, 15-keto-PGE_2_, PGA_2_, as well as the hydroxylated fatty acid derivatives 8-HETE and leukotriene B_4_ have previously been extracted from *G. vermiculophylla* [40]–[42]. In our experiments we detected also prostaglandins and 8-HETE involved in the metabolic response to wounding. In addition, 7,8-di-HETE and a novel conjugated lactone were found to be highly up-regulated in wounded tissue. The results could be partly confirmed in GC/MS studies, but there the responses were not as pronounced, presumably due to the low stability of the eicosanoids during derivatisation, injection and separation. Contrary to higher plants, which generally do not produce derivatives of arachidonic acid [43], eicosanoids have been documented for a number of marine macroalgae [44], [45]. Such eicosanoids are well known as metabolites regulating cell differentiation, immune responses and homeostasis in animal systems [46]. The role of eicosanoids in macroalgae is not well understood, but a few studies suggest that they are parts of their innate defence system as well [6], [33], [47], [48]. Although free arachidonic acid was detected in *G. vermiculophylla*, we did not detect elevated levels in wounded tissue, possibly due to a rapid oxygenation of the molecule. This is confirmed by experiments where stable isotope labelled arachidonic acid is administered to wounded algal tissue. The labelled fatty acids were readily converted to the prostaglandins, the hydroxylated and dihydroxylated C20 fatty acids as well as in the conjugated lactone (Fig. 4, incorporation rates given in Table 2). This metabolic response is remarkable, since apparently the entire enzymatic machinery required to produce prostaglandins and hydroxylated fatty acids is active in the wounded algae. The up-regulation of the hydroxylated eicosanoids 8-HETE and 7,8-di-HETE in mechanically wounded tissue suggests that *G. vermiculophylla* responds to tissue damage similarly as the closely related *Gracilaria chilensis* [33]. This alga releases free arachidonic acid, 8-HETE and 7,8-di-HETE that are involved in the defence against biofouling, it does however not produce elevated amounts of prostaglandins. The release of free arachidonic acid and subsequent formation of 8-HETE in *G. chilensis* was shown to be controlled by phospholipase A, while 7,8-di-HETE was directly released from galactolipid [33]. Incorporation rates with *G. vermiculophylla* however prove that this alga can also produce 7,8 di-HETE in response to wounding from arachidonic acid (Table 2). Both *Gracilaria* species are thus relying on wound activated production of C20 oxylipins but exploit fundamentally different mechanisms resulting in different metabolic patterns as well as in a different use of resources.

We tested the involvement of the up-regulated metabolites in bioassays with *I. baltica* using artificial food containing single metabolites or mixtures. Natural concentrations of arachidonic acid derived metabolites in wounded algae are in the concentration range of 1-13 µg g^-1^ algae (fresh weight). The relative and absolute concentrations vary significantly, most likely due to biological variability of the algal material and the poor reproducibility of the wounding procedure. We therefore tested concentration ranges of the respective metabolites (Fig. 1B). The prostaglandin PGA_2_ exhibited a significant concentration dependent defensive potential against *I. baltica*, supporting the role of the eicosanoids as parts of the innate defence system in marine macroalgae. Even if this prostaglandin showed up as only a minor metabolite in our analysis (Fig. 3, Table 2) it might not have been detected in higher quantities due to its limited stability. This has been confirmed in later studies using an extraction protocol optimized for this compound class where levels of PGA_2_ higher than 0.6 µg g^−1^ algal wet weight, which we found in this study, have repeatedly been detected in wounded algal samples (Hammann et al. unpublished). This observation reflects a known limitation of metabolomics, a technique designed to pick up as many metabolites as possible, which is not optimised for specific compound classes. In contrast, all other metabolites tested were virtually inactive or even rendered the artificial food more attractive. It is remarkable to observe that the isomer 8-iso-PGA_2_ of PGA_2_, which only differs in the relative configuration of the side chains, is inactive even at elevated concentrations. The defensive potential of metabolites is thus strongly structure dependent, indicating the requirement for a specific structure activity relation. A mixture of the most abundant up-regulated natural oxylipins (PGA_2_ : PGE_2_ : 15-keto-PGE_2_ : 7,8-di-HETE 1∶1∶1∶0.5) tested at concentrations also found in wounded algal tissue was not more active than PGA_2_ tested at corresponding concentration in isolation, which suggests that PGA_2_ is solely responsible for the grazing deterrent effect. However, given the high specificity of defence potential of the metabolites it can be envisaged, that hitherto uncharacterized or untested metabolites contribute in addition to the chemical defence of the alga.

Although all herbivory results in plant tissue damage, tissue disruption per se is not always a reliable indicator of grazer attack [39], [49]. In this study, herbivory was simulated by mechanical wounding and it can be argued that this caused a reaction different from direct grazing. However, direct grazing of *I. baltica* caused a similar metabolic response as mechanical wounding. This shows that the observed defence mechanisms in *G. vermiculophylla*, elicited by mechanical wounding, also are stimulated by *I. baltica* feeding, and thus indeed occur in response to herbivory.

In the LC/MS investigations where we monitored for the induced defence of *G. vermiculophylla* we observed only a minor up-regulation of one metabolite that was also up-regulated in the metabolic response to wounding. All other metabolites that show a pronounced response to wounding are not affected by the induction protocol. In contrast a pronounced change in the metabolic profile monitored with GC/MS was observed upon induction (Fig. 2D). Analyses of GC/MS data showed that 44 metabolites were significantly up-regulated in algae exposed to extracts from mechanically wounded tissue. Comparisons with the NIST library and co-injections with known standards suggested that a considerable part of the up-regulated metabolites were primary metabolites, such as amino acids, saccharides, sterols and fatty acids, which indicates a general stress response in induced algae [48], [50]–[52]. These compounds were not tested in bioassays and thus the identification of the true induced defensive metabolites still remains open. Alternatively, the poor palatability of the induced algae might be explained with a reduced food quality as a result of changed levels of primary metabolites. Studies investigating hormonal activity in marine macroalgae are rare [47], [51], [53], and an algal hormone is still to be identified. Results from our study again illustrate the difference of the defence response of *G. vermiculophylla* in comparison to the related *G. chilensis* where an induced defence against epiphytes could be triggered by an oxylipin enriched fraction, but the metabolic response included the up-regulation of lipases, lipoxygenases and the de novo production of eicosanoids [6]. The fact that an eicosanoid rich extract is capable to induce chemical defence and to regulate multiple metabolic pathways in both algae suggests that they rely on defence hormone(s) as it is known from higher plants.

It has been predicted that well-defended exotics are most likely to become successful invaders in systems such as marine macroalgal communities that are dominated by generalist grazers exerting a strong selection pressure [29]. The underlying logic is that generalist enemies, which by definition are prone to host switching and/or diet mixing, should accumulate on potential invaders compared to native species that are likely to have evolved effective defences against the coexisting generalist enemies [54]. Such selective attack of exotic plants would select for well-defended exotic species or genotypes. A number of studies have shown that high defence levels can be a successful strategy for invaders in systems dominated by generalist herbivores [29], [55], [56], [57]. However, a recent survey of 19 invasive plants and 21 co-occurring native plants shows that although particular invasive species may possess deterrent secondary metabolites chemical deterrence does not seem to be a general feature for invasive species [58]. Thus, the described chemical defence against herbivory in *G. vermiculophylla* may be one of several possible explanations to the rapid expansion of *G. vermiculophylla* into new areas, although it was beyond the scope of this study to explain mechanisms of invasion success.

In conclusion, this study suggests that *G. vermiculophylla* chemically deters herbivory by a rapid wound-activated defence followed by an inducible defence. The use of a metabolomics approach enabled a straightforward detection of defence metabolites and provided novel information about the biochemical pathway involved in the defence.

## Materials and Methods

### Study organisms and collections

*G. vermiculophylla* is a perennial red macroalga native to the Northwest Pacific [24]. It has an isomorphic lifestyle with male and female gametophytes and sporophytes of similar morphology (Ohmi 1956). *G. vermiculophylla* typically grows shallow as loose-lying thalli or attached to small stones and mollusc shells within low-energy bays and estuaries [28], [30]. For chemical investigations and bioassays, algal specimens were collected from the Kiel Bay, in the Baltic, the Great Belt, and Horsens Fjord. Several collections were made during the study.

The palatability of algae and algal extracts was assessed with bioassays using the crustacean *I. baltica*. Individuals of *I. baltica* were collected from seaweeds in the Kiel Bay and maintained in the laboratory in a flow-through system with natural seawater until used in the bioassays. The herbivores were offered mixtures of various seaweeds as diets. No specific permits were required for the collection and experimentation of the study organisms.

### Mechanical wounding and intense grazing of algal thalli

Fresh algal material was blotted dry with precision wipes and 20 samples (200 mg wet weight) were prepared. To monitor the metabolic profile in intact tissue, 10 samples (n = 10) were shock frozen in liquid nitrogen, ground to a fine powder and the cold powder was extracted in 3 ml methanol (MeOH) for 5 h on a vortex. Herbivory was mechanically simulated by grinding the other 10 samples in a mortar and subsequent incubation for 5 min at room temperature before MeOH extraction as described above. After extraction, the samples were centrifuged to remove solid material and stored at −20°C until chemical analyses. Additional extractions of totally 20 g wet weight of each mechanically wounded and control algae were made as above to prepare extracts for bioassays and induction experiments.

In order to investigate if natural grazing causes a similar metabolic response as mechanical simulation of herbivory high densities of *I. baltica* (8 individuals per 100 mg alga) were allowed to graze on *G. vermiculophylla* samples (n = 8) for 1 and 2 h, before the algal samples were frozen in liquid nitrogen and extracted as above. The algae were weighed before and after the treatment to calculate grazer consumption. Algal samples incubated for 1 and 2 h without grazers served as controls. After extraction and centrifugation to remove solid material, the metabolites significantly up- and down regulated following mechanical simulation of herbivory were measured in the samples.

### Induction experiment

*G. vermiculophylla* (2.5 g, n = 7) were incubated for 7 h in 50 ml seawater containing 5 mg MeOH-extract from mechanically wounded or control algae that was evaporated to dryness and dissolved in 0.5 ml DMSO. The final concentration corresponded to the amount of extract from approximately 150 mg algal tissue in 50 ml seawater. After incubation, the algae were transferred to aquaria of 2.5 l volume with running seawater (1 l h^−1^) at ambient water temperature and a light:dark cycle of 10∶14 h. After 4 d of incubation, the algae were removed, freeze-dried, individually ground to a fine powder of which 50 mg was extracted in 3 ml MeOH for 5 h on a vortex. The resulting extracts were centrifuged to remove solid material and stored at −20°C until chemical analysis. The rest of the algal powder was used for bioassays.

### Neighbouring effect of local mechanical wounding

Unbranched filaments of equal sizes (length 4 cm) were isolated from fresh specimens of *G. vermiculophylla* (n = 8). The tips of the filaments (0.5 cm) were wounded with forceps and the algal samples were incubated in seawater for 1 h at room temperature. After the incubation, 1 cm of the tips (0.5 cm damaged and 0.5 cm undamaged tissue) was cut and the remaining parts were shock frozen in liquid nitrogen and ground to a fine powder before extraction with cold MeOH. Control algae with undamaged tips were also incubated for 1 h and 1 cm of the tips were collected according to the above mentioned procedure to control for possible cutting effects. Thereafter, the control samples were extracted the same way as the wounded samples. After extraction and centrifugation to remove solid material, the metabolites significantly up- and down regulated following mechanical simulation of herbivory were measured in the samples.

### Herbivore bioassays

Two-way-choice feeding assays tested the feeding behaviour of the isopod *I. baltica* using agar-based artificial food as described previously [59]. Briefly, two different (“control” and “treatment”) food pellets containing 10 % freeze-dried algal biomass, 3.6 % agar and mosquito gauze as a uniform internal grid were offered in a Petri dish to two individuals of *I. baltica*, which were allowed to consume *ad libitum* at 15°C. The consumption of both pellets during up to 3 d was recorded repeatedly as the number of squares of the gauze that had been cleared. To test if exposure of intact *G. vermiculophylla* to extracts from wounded algae causes increased resistance against grazing, *G. vermiculophylla* powder from induced and uninduced plants was offered to *Idotea baltica* (n = 7, see above). This experiment was run for 40 h, although the data used for the statistical analysis came mostly from readings after 17 and 24 h. To test for the effect of mechanical simulation of herbivory on palatability, all pellets were made up with a batch of freeze-dried *Ulva lactuca* biomass. Treatment pellets were charged with MeOH-extract of wounded *G. vermiculophylla* redissolved in DMSO, while control pellets contained extract of intact *G. vermiculophylla* (n = 10, see above). All extracts were dosed at natural concentration, based on the assumption of a dry matter content in seaweeds of 10 %, i.e., pellet containing 0.5 g *U. lactuca* dry matter in 5 ml H_2_O was charged with extract yielding from 5 g wet weight *G. vermiculophylla* and redissolved in 250 µl DMSO. In this experiment readings after 40 h of incubation with *I. baltica* were used for the statistical analysis. For the testing of pure metabolites, *U. lactuca* biomass was also used as the main component of food pellets, while metabolites dissolved in MeOH (acetonitrile in the case of 7,8-di-HETE) were employed at concentrations between 0.2 and 20 µg g^−1^ of algal wet weight and tested against solvent only as a control (n = 6).

For data evaluation the ratio of eaten and uneaten mesh squares of the control pellet was divided by the ratio of eaten and uneaten mesh squares in the treatment pellet as described previously [59]. The resulting odds ratio ω represents the approximate risk of preferential feeding of the control pellet. No feeding preference is given if ω = 1, while ω<1 indicates a preference for the treatment pellet and ω>1 a preference for the control pellet. The divergence of ω from 1 was tested using Fisher's exact test.

### Non-targeted metabolic profiling

Metabolic profiling was performed using LC/MS and GC/MS analyses. For LC/MS analysis, MeOH extracts prepared as described above were injected into a Waters Acquity ultra-performance liquid chromatography (UPLC, Waters, Manchester, UK) coupled to a time of flight Q-TOF micro mass spectrometer (Waters) equipped with electrospray ionisation (ESI). Mass spectra were recorded in negative ion mode. A 5 cm BEH C18 UPLC column (2.1 mm, 1.7 µm; Waters) at 30°C was used for separation. The flow rate was 0.6 ml min^–1^ and the mobile phases were 0.1 % formic acid and 2 % acetonitrile in water ( = A), and 0.1 % formic acid in acetonitrile ( = B). The solvent gradient was: 100 % A to 100 % B in 7 min, 100 % B for 2 min, 100 % B to 100 % A in in 30 s, 100 % A for 30 s (all solvents ULC/MS grade, Biosolve, Valkenswaard, the Netherlands). Mass-spectra were recorded at a scan rate of 1 scan s^–1^ with an inter scan delay of 0.1 s and a scan range of 100 to 1000 *m/z*. The collision energy was 5 V, the sample cone voltage was 25 V, the cone gas flow was 50 l N_2_ h^−1^ and the desolvation gas flow was 650 l N_2_ h^−1^. Quantification of up-regulated metabolites was performed after calibration with external standards. Calibration substances were either commercial available (compound 2, 3 and 4, Cayman Chemicals, Ann Arbor, MI, USA) or isolated from extracts that were accredited by GC/MS, LC/MS and NMR. The most abundant pseudomolecular ion trace of the respective metabolites was used for quantification.

For GC/MS analysis, 100 µl MeOH extracts prepared as described above were evaporated and derivatised by methoxyamination and subsequent trimethylsilylation as described previously [60]. A Waters GCT premier (Waters, Manchester, UK) coupled to an Agilent 6890N GC equipped with a DB-5 ms 30 m column (0.25 mm internal diameter, 0.25 µm film thickness, Agilent, USA) and a 10 m duraguard guard column was used for GC/MS measurements. The inlet temperature was maintained at 280°C and the column held at 60°C for 1 min, ramped at 15°C min^−1^ to 310°C and held at this temperature for 10 min. Helium was used as carrier gas at a constant flow of 1 ml min^−1^ and the transfer line was held at 250°C. Ionisation energy was 70 eV with the ion source at 250°C. The TOF mass detector was operated at a scan rate of 2 scans s^−1^ with an inter scan delay of 0.05 s and a scan range of 20 to 1000 *m/z* using the dynamic range extension mode.

### Chemometric analysis

LC/MS and GC/MS raw data were analysed using the MarkerLynx V4.1 software (Waters). This application integrates peaks using ApexTrack peak detection. Lists of peak intensities were generated using retention time (RT) and *m/z* data pairs as the identifier for each peak.

This process was repeated for each run in the batch and the data were combined by aligning peaks with the same RT - *m/z* pair from each data file in the data set. For data binning, retention time windows of 0.4 min and *m/z* windows of 0.5 Da were allowed as tolerance, and peaks within this window were considered to be identical. The resulting 3-dimensional data, RT - *m/z* pair, sample name and ion intensity were analysed with canonical analysis of principal coordinates (CAP), using range-transformed data normalised to the amount of extracted material and the Bray-Curtis distance measure [61]–[63]. CAP is a constrained ordination that accounts for the information inherent in the experimental design by finding the canonical axis that maximises the separation among treatment and control samples. Correlations between *m/z*-retention time pairs and canonical axis represent the individual contribution of a *m/z*-retention time pair to the axis. Fragmentation of molecules in GC/MS and to some extent LC/MS, as well as natural isotopes results in several RT - *m/z* pairs per unique molecule. Such RT - *m/z* pairs were combined into arbitrarily named metabolites by visual inspection of mass spectra and retention times. This was performed with the RT - *m/z* pairs that were significantly correlated to the canonical axes. The peak areas of the obtained metabolites were then analysed with permutational multivariate analysis of variance (PERMANOVA) and CAP, using the Canberra distance measure to create the matrix of dissimilarities between objects [62]–[65].

To graphically represent the relationships between the samples, principal coordinates analysis with range-transformed data and the Bray-Curtis distance measure was used [61].

### Characterisation of up-regulated metabolites

We used results from the metabolomic studies to identify up-regulated signals that were selected for further structure elucidation. Co-injections with known standards formed the basis for characterisation of known metabolites. The choice of standards was based on a combination of studies of mass spectra and retention times, comparisons of spectral data with spectra in the U.S. National Institute of Standards and Technology (NIST) library (only GC/MS analyses) and previous chemical studies on *G. vermiculophylla* and *G. chilensis* [33], [40]–[42]. For oxylipins identified in LC/MS studies ^1^H and ^13^C NMR data were collected from purified fractions for verification of the structures. Eight known eicosanoids and fatty acids were used to characterise LC/MS detected up-regulated metabolites, and a mixture of totally 56 amino acids, fatty acids, fatty alcohols, sterols and sugars were used to characterise GC/MS detected up-regulated metabolites.

### Biosynthesis of oxylipins

To test if up-regulated metabolites are produced de-novo from arachidonic acid we administered 2[^2^H_1_]- and 2[^2^H_2_]-arachidonic acid following an established procedure [66]. The respective mono or dideuterated fatty acids (2 mg suspended in 15 µl water) were added to 10 mg of algal sample that was frozen in liquid nitrogen and ground to a fine powder. The mixture was vortexed and allowed to warm to room temperature. After 10 minutes 300 µl of MeOH/water (2∶1 v/v) was added. After mixing and centrifugation, the supernatant was used directly for LC/MS analysis. Incorporation rates were determined by evaluating the integrals of the ion traces of labeled and non-labeled metabolites.

## References

[1] Rosenthal G, Berenbaum MR (1992). Herbivores: their interactions with secondary metabolites..

[2] Paul VJ, Cruz-Rivera E, Thacker RW (2001). Chemical mediation of macroalgal-herbivor interactions: ecological and evolutionary perspectives. In: McClintock, JB, Baker, BJ (eds) Marine Chemical Ecology.. CRC Press, Boca Raton, FL,.

[3] Karban R, Baldwin I (1997). Induced responses to herbivory..

[4] Toth GB, Pavia H (2007). Induced herbivore resistance in seaweeds: a meta-analysis.. J Ecol.

[5] Weinberger F (2007). Pathogen-induced defence and innate immunity in macroalgae..

[6] Weinberger F, Lion U, Delage L, Kloareg B, Potin P (2011). Up-regulation of lipoxygenase, phospholipase, and oxylipin-production in the induced chemical defense of the red alga *Gracilaria chilensis* against epiphytes.. J Chem Ecol.

[7] Paul VJ, Van Alstyne KL (1992). Activation of chemical defenses in the tropical green algae *Halimeda* spp.. J Exp Mar Biol Ecol.

[8] Wittstock U, Gershenzon J (2002). Constitutive plant toxins and their role in defense against herbivores and pathogens.. Curr Opin Plant Biol.

[9] Cetrulo GL, Hay ME (2000). Activated chemical defenses in tropical versus temperate seaweeds.. Mar Ecol Prog Ser.

[10] Pohnert G (2004). Chemical defence strategies of marine organisms.. The chemistry of pheromones and other semiochemicals.

[11] Baldwin IT, Callahan P (1993). Autotoxicity and chemical defence - nicotine accumulation and carbon gain in solanaceous plants.. Oecologia.

[12] Wolfe GV, Steinke M, Kirst GO (1997). Grazing-activated chemical defence in a unicellular marine alga.. Nature.

[13] Hay ME, Pawlik JR, Duffy JE, Fenical W (1989). Seaweed-herbivore-predator interactions - host-plant specialization reduces predation on small herbivores.. Oecologia.

[14] Giamoustaris A, Mithen R (1995). The effect of modifying the glucosinolate content of leaves of oilseed rape (*Brassica napus* ssp. *oleifera*) on its interaction with specialist and generalist pests.. Ann Appl Biol.

[15] Honda K, Hayashi N, Abe F, Yamauchi T (1997). Pyrrolizidine alkaloids mediate host-plant recognition by ovipositing females of an old world danaid butterfly, *Idea leuconoe*.. J Chem Ecol.

[16] Coleman RA, Ramchunder SJ, Moody AJ, Foggo A (2007). An enzyme in snail saliva induces herbivore-resistance in a marine alga.. Funct Ecol.

[17] Nathanson JA (1984). Caffeine and related methylxanthines - possible naturally-occurring pesticides.. Science.

[18] Barbosa P, Saunders JA, Kemper J, Trumbule R, Olechno J (1986). Plant allelochemicals and insect parasitoids effects of nicotine on *Cotesia congregata* (Say) (Hymenoptera, Braconidae) and *Hyposoter annulipes* (Cresson) (Hymenoptera, Ichneumonidae).. J Chem Ecol.

[19] Hay ME, Duffy JE, Pfister CA (1987). Chemical defense against different marine herbivores: are amphipods insect equivalents?. Ecology.

[20] Weinberger F, Pohnert G, Kloareg B, Potin P (2002). A signal released by an enclophytic attacker acts as a substrate for a rapid defensive reaction of the red alga *Chondrus crispus*.. ChemBioChem.

[21] Taylor RB, Lindquist N, Kubanek J, Hay ME (2003). Intraspecific variation in palatability and defensive chemistry of brown seaweeds: effects on herbivore fitness.. Oecologia.

[22] Bundy JG, Davey MP, Viant MR (2009). Environmental metabolomics: a critical review and future perspectives.. Metabolomics.

[23] Prince EK, Pohnert G (2010). Searching for signals in the noise: metabolomics in chemical ecology.. Anal Bioanal Chem.

[24] Tseng C, Xia B (1999). On the Gracilaria in the western Pacific and the southeastern Asia region.. Bot Mar.

[25] Rueness J (2005). Life history and molecular sequences of *Gracilaria vermiculophylla* (Gracilariales, Rhodophyta), a new introduction to European waters.. Phycologia.

[26] Freshwater DW, Montgomery F, Greene JK, Hamner RM, Williams M (2006). Distribution and identification of an invasive *Gracilaria* species that is hampering commercial fishing operations in southeastern North Carolina, USA.. Biol Invasions.

[27] Thomsen M, Staehr P, Nyberg C, Schwærter S, Krause-Jensen D (2007). *Gracilaria vermiculophylla* (Ohmi) Papenfuss, 1967 (Rhodophyta, Gracilariaceae) in northern Europe, with emphasis on Danish conditions, and what to expect in the future.. Aquat Invasions.

[28] Nyberg CD, Thomsen MS, Wallentinus I (2009). Flora and fauna associated with the introduced red alga *Gracilaria vermiculophylla*.. Eur J Phycol.

[29] Wikström SA, Steinarsdottir MB, Kautsky L, Pavia H (2006). Increased chemical resistance explains low herbivore colonization of introduced seaweed.. Oecologia.

[30] Weinberger F, Buchholz B, Karez R, Wahl M (2008). The invasive red alga *Gracilaria vermiculophylla* in the Baltic Sea: adaptation to brackish water may compensate for light limitation.. Aquat Biol.

[31] Engkvist R, Malm T, Tobiasson S (2000). Density dependent grazing effects of the isopod *Idotea baltica* Pallas on *Fucus vesiculosus* L in the Baltic Sea.. Aquat Ecol.

[32] Engkvist R, Malm T, Nilsson J (2004). Interaction between isopod grazing and wave action: a structuring force in macroalgal communities in the southern Baltic Sea.. Aquat Ecol.

[33] Lion U, Wiesemeier T, Weinberger F, Beltran J, Flores V (2006). Phospholipases and galactolipases trigger oxylipin-mediated wound-activated defence in the red alga *Gracilaria chilensis* against epiphytes.. ChemBioChem.

[34] Howe GA, Jander G (2008). Plant immunity to insect herbivores.. Annu Rev Plant Biol.

[35] Boller T, He SY (2009). Innate immunity in plants: an arms race between rattern recognition receptors in plants and effectors in microbial pathogens.. Science.

[36] Grant MR, Jones JDG (2009). Hormone (dis)harmony moulds plant health and disease.. Science.

[37] Toth GB (2007). Screening for induced herbivore resistance in Swedish intertidal seaweeds.. Mar Biol.

[38] Cronin G, Hay ME (1996). Induction of seaweed chemical defenses by amphipod grazing.. Ecology.

[39] Pavia H, Toth GB (2000). Inducible chemical resistance to herbivory in the brown seaweed *Ascophyllum nodosum*.. Ecology.

[40] Sajiki J (1999). Acid treatment increased leukotriene B-4 in the red alga, *Gracilaria asiatica* ( = *verrucosa*).. Fisheries Sci.

[41] Sajiki J (1997). Effects of acetic acid treatment on the concentrations of arachidonic acid and prostaglandin E(2) in the red algae, *Gracilaria asiatica* and *G.rhodocaudata*.. Fisheries Sci.

[42] Sajiki J, Kakimi H (1998). Identification of eicosanoids in the red algae, *Gracilaria asiatica*, using high-performance liquid chromatography and electrospray ionization mass spectrometry.. J Chromatogr A.

[43] Weber H (2002). Fatty acid-derived signals in plants.. Trends Plant Sci.

[44] Guschina IA, Harwood JL (2006). Lipids and lipid metabolism in eukaryotic algae.. Prog Lipid Res.

[45] Andreou A, Brodhun F, Feussner I (2009). Biosynthesis of oxylipins in non-mammals.. Prog Lipid Res.

[46] Funk CD (2001). Prostaglandins and leukotrienes: Advances in eicosanoid biology.. Science.

[47] Bouarab K, Adas F, Gaquerel E, Kloareg B, Salaun JP (2004). The innate immunity of a marine red alga involves oxylipins from both the eicosanoid and octadecanoid pathways.. Plant Physiol.

[48] Ritter A, Jean-Pierre Sala S, Tonon T, Correa J, Potin P (2008). Copper stress induces biosynthesis of octadecanoid and eicosanoid oxygenated derivatives in the brown algal kelp *Laminaria digitata*.. New Phytol.

[49] Dicke M, van Poecke RMP, de Boer JG (2003). Inducible indirect defence of plants: from mechanisms to ecological functions.. Basic Appl Ecol.

[50] Kupper FC, Gaquerel E, Boneberg EM, Morath S, Salaun JP (2006). Early events in the perception of lipopolysaccharides in the brown alga *Laminaria digitata* include an oxidative burst and activation of fatty acid oxidation cascades.. J Exp Bot.

[51] Gaquerel E, Herve C, Labriere C, Boyen C, Potin P (2007). Evidence for oxylipin synthesis and induction of a new polyunsaturated fatty acid hydroxylase activity in *Chondrus crispus* in response to methyljasmonate.. Biochim Biophys Acta, Mol Cell Biol Lipids.

[52] Kakinuma M, Coury DA, Kuno Y, Itoh S, Kozawa Y (2006). Physiological and biochemical responses to thermal and salinity stresses in a sterile mutant of *Ulva pertusa* (Ulvales, Chlorophyta).. Mar Biol.

[53] Collen J, Herve C, Guisle-Marsollier I, Leger JJ, Boyen C (2006). Expression profiling of *Chondrus crispus* (Rhodophyta) after exposure to methyl jasmonate.. J Exp Bot.

[54] Colautti RI, Ricciardi A, Grigorovich IA, MacIsaac HJ (2004). Is invasion success explained by the enemy release hypothesis?. Ecol Lett.

[55] Amade P, Lemee R (1998). Chemical defence of the Mediterranean alga *Caulerpa taxifolia*: variations in caulerpenyne production.. Aquat Toxicol.

[56] Joshi J, Vrieling K (2005). The enemy release and EICA hypothesis revisited: incorporating the fundamental difference between specialist and generalist herbivores.. Ecol Lett.

[57] Schaffner U, Ridenour WM, Wolf VC, Bassett T, Müller C, Müller-Schärer H, Sutherland S, Lortie CJ, Callaway RM (2011). Plant invasions, generalist herbivores, and novel defense weapons.. Ecology.

[58] Lind EM, Parker JD (2010). Novel weapons testing: are invasive plants more chemically defended than native plants?. PLoS ONE.

[59] Weinberger F, Rohde S, Oschmann Y, Shahnaz L, Dobretsov S (2011). Effects of limitation stress and of disruptive stress on induced antigrazing defense in the bladder wrack *Fucus vesiculosus*.. Mar Ecol Prog Ser.

[60] Lisec J, Schauer N, Kopka J, Willmitzer L, Fernie A (2006). Gas chromatography mass spectrometry-based metabolite profiling in plants.. Nat Protoc.

[61] Legendre P, Legendre L (1998). Numerical Ecology, 2nd English edn..

[62] Anderson MJ, Willis TJ (2003). Canonical analysis of principal coordinates: A useful method of constrained ordination for ecology.. Ecology.

[63] Anderson MJ (2004). CAP: A FORTRAN computer program for canonical analysis of principal coordinates..

[64] Anderson MJ (2001). A new method for non-parametric multivariate analysis of variance.. Austral Ecol.

[65] Anderson MJ (2005). PERMANOVA: A FORTRAN computer program for permutational multivariate analysis of variance..

[66] Rempt M, Pohnert G (2010). Novel acetylenic oxylipins from the moss *Dicranum scoparium* with antifeeding activity against herbivorous slugs.. Angew Chem Int Edit.

